# Exploring the neuroprotective effects of montelukast on brain inflammation and metabolism in a rat model of quinolinic acid-induced striatal neurotoxicity

**DOI:** 10.1186/s12974-023-02714-z

**Published:** 2023-02-13

**Authors:** Margherita Tassan Mazzocco, Valentina Murtaj, Daniel Martins, Roberta Schellino, Angela Coliva, Elisa Toninelli, Alessandro Vercelli, Federico Turkheimer, Sara Belloli, Rosa Maria Moresco

**Affiliations:** 1 PhD Program in Neuroscience, Medicine and Surgery Department, University of Milano-Bicocca, Milan, Italy; 2 Nuclear Medicine Department, San Raffaele Scientific Institute (IRCCS), Milan, Italy; 3 Department of Neuroimaging, Institute of Psychiatry, Psychology and Neuroscience, King’s College London, London, UK; 4 Department of Neuroscience “Rita Levi Montalcini” and Neuroscience Institute Cavalieri Ottolenghi, University of Turin, Turin, Italy; 5 Institute of Molecular Bioimaging and Physiology (IBFM), CNR, Milan, Italy; 6 Technomed Foundation and Department of Medicine and Surgery, University of Milano-Bicocca, Milan, Italy

**Keywords:** Montelukast, Neuroinflammation, Positron emission tomography, Quinolinic acid, Neurodegeneration, Cysteinyl leukotriene receptor antagonist, Metabolic connectivity, 18-kDa translocator protein, Medium spiny neurons

## Abstract

**Background:**

One intrastriatal administration of quinolinic acid (QA) in rats induces a lesion with features resembling those observed in Huntington’s disease. Our aim is to evaluate the effects of the cysteinyl leukotriene receptor antagonist montelukast (MLK), which exhibited neuroprotection in different preclinical models of neurodegeneration, on QA-induced neuroinflammation and regional metabolic functions.

**Methods:**

The right and left striatum of Sprague Dawley and athymic nude rats were injected with QA and vehicle (VEH), respectively. Starting from the day before QA injection, animals were treated with 1 or 10 mg/kg of MLK or VEH for 14 days. At 14 and 30 days post-lesion, animals were monitored with magnetic resonance imaging (MRI) and positron emission tomography (PET) using [^18^F]-VC701, a translocator protein (TSPO)-specific radiotracer. Striatal neuroinflammatory response was measured post-mortem in rats treated with 1 mg/kg of MLK by immunofluorescence. Rats treated with 10 mg/kg of MLK also underwent a [^18^F]-FDG PET study at baseline and 4 months after lesion. [^18^F]-FDG PET data were then used to assess metabolic connectivity between brain regions by applying a covariance analysis method.

**Results:**

MLK treatment was not able to reduce the QA-induced increase in striatal TSPO PET signal and MRI lesion volume, where we only detected a trend towards reduction in animals treated with 10 mg/kg of MLK. Post-mortem immunofluorescence analysis revealed that MLK attenuated the increase in striatal markers of astrogliosis and activated microglia in the lesioned hemisphere. We also found a significant increase in a marker of anti-inflammatory activity (MannR) and a trend towards reduction in a marker of pro-inflammatory activity (iNOS) in the lesioned striatum of MLK—compared to VEH-treated rats. [^18^F]-FDG uptake was significantly reduced in the striatum and ipsilesional cortical regions of VEH-treated rats at 4 months after lesion. MLK administration preserved glucose metabolism in these cortical regions, but not in the striatum. Finally, MLK was able to counteract changes in metabolic connectivity and measures of network topology induced by QA, in both lesioned and non-lesioned hemispheres.

**Conclusions:**

Overall, MLK treatment produced a significant neuroprotective effect by reducing neuroinflammation assessed by immunofluorescence and preserving regional brain metabolism and metabolic connectivity from QA-induced neurotoxicity in cortical and subcortical regions.

**Supplementary Information:**

The online version contains supplementary material available at 10.1186/s12974-023-02714-z.

## Background

Huntington's disease (HD) is an autosomal dominant genetic disorder caused by a CAG triplet expansion in the huntingtin gene (HTT), encoding an expanded polyglutamine (polyQ) tract near the N-terminus of the HTT protein. This mutation induces a progressive neurodegenerative disease characterized by motor symptoms including chorea and dystonia, as well as psychiatric and cognitive abnormalities [1]. In the early phases, HD pathology is characterized by neuronal loss, particularly of the medium-sized projection neurons of the dorsal striatum, which then spreads to cortical and sub-cortical regions [2].

Several post-mortem and clinical studies highlighted a clear role of microglia and astrocytes in the pathogenesis and progression of HD. In particular, the accumulation of mutated HTT (mHTT) in neurons stimulates the NFκB pathway, leading to microglia activation and pro-inflammatory cytokines release [3–5]. Moreover, mHTT accumulates also in the astrocytes, where it promotes oxidative stress and reduces BDNF levels [6–8]. In line with these post-mortem findings, human positron emission tomography (PET) studies using ligands for the translocator protein (TSPO), a widely used marker for microglia activation, showed that neuroinflammation is present in pre-symptomatic HD gene carriers, suggesting that microglial activation starts at early stages of HD [9, 10]. In HD patients, TSPO binding increases in striatal and cortical regions where it positively correlates with disease severity [11]. For these reasons, the modulation of neuroinflammation represents an attractive strategy for the development of novel HD treatments.

Montelukast (MLK) is a cysteinyl leukotriene receptor 1 (CysLT1R) and G protein-coupled receptor 17 (GPR17) antagonist, approved as adjuvant therapy for asthma in both children and adults [12–14]. Previous studies have reported these receptors to be involved in different neurological disorders [15–19]. Furthermore, increased levels of leukotrienes have been found after injuries of the central nervous system (CNS) and during aging [20, 21], where they are thought to play an important role in modulating the neuroinflammatory response. For these reasons, cysteinyl leukotriene receptors (CysLTRs) have been proposed as a potential drug repurposing target for conditions associated with neuroinflammation and neurodegeneration [22].

Different lines of evidence from preclinical models of neurological disorders, including Alzheimer’s disease, Parkinson’s disease, ischemia–reperfusion and aging concur to support the ideas that MLK exerts a neuroprotective effect, including preservation and restoration of the blood–brain barrier (BBB) [23], neurogenesis stimulation [24], reduction of inflammation, oxidative stress and apoptosis together with amelioration of brain functions and cognition [25–33].

Based on these accumulated data in preclinical models of neurodegeneration, it is reasonable to hypothesize that MLK might similarly have neuroprotective effects in HD by attenuating the neuroinflammatory response which accompanies the disease. However, no studies to date have comprehensively tested this hypothesis. In this study, we sought to bridge this gap by investigating the effects of MLK treatment on the brain pathology underlying HD using intrastriatal injection of quinolinic acid (QA) in rats. Intrastriatal injection of QA in rats has been demonstrated to induce neuropathological and behavioral alterations whose features and pattern of time progression resemble those observed in patients with HD [34–36]. QA is an excitotoxin that selectively stimulates NMDA receptors (NMDAR) and, when in high levels, it is able to induce neurodegeneration, particularly in NMDAR-expressing neurons. QA also affects astrocytes, reducing their buffering effects on glutamate levels and increasing the inflammatory response to brain insults (see Guillemin 2012 for a review on QA effects [37]). Moreover, QA is an endogenous downstream product of the kynurenine pathway (KP) and abnormalities in KP metabolism in microglia and astrocytes may explain the increased levels of QA found in the neostriatum and cortex of early-stage HD patients favoring neurodegeneration [38, 39]. Altogether, these data support the validity of the QA model as a tool to study the brain pathology underlying HD and the effects of putative innovative disease-modifying drugs. To date, only one study has investigated the effects of MLK on the QA model, showing that MLK attenuated behavioral impairments, oxidative stress and mitochondrial dysfunctions [40]. However, this study did not thoroughly investigate the effects of the drug on the neuroinflammatory response, which is likely to play a role in the beneficial effects of MLK in this HD model. Therefore, in this work, we took a multimodal approach to explore the effects of MLK on QA-lesioned brain tissue using a combination of in vivo PET, Magnetic Resonance Imaging (MRI) and post-mortem immunofluorescence. In particular, we evaluated the neuroprotective effect of MLK treatment on tissue damage, neuroinflammation and brain metabolic connectivity in the QA unilateral cytotoxic rat model. Since the role of T-cells in brain response to neurotoxins has been recently proposed [41], this study was performed on both immunocompetent and athymic nude rats.

## Material and methods

### Animals

Twenty-four Sprague Dawley (SD) and 30 athymic NIH FOXN1-RNU nude male rats, approximately 6 weeks old (mean body weight: 180 g) were purchased from ENVIGO RMS SRL (Italy). Animals were housed in the San Raffaele Scientific Institute animal facility and maintained under constant temperature and humidity conditions, with a 12/12-h light/dark cycle and access to food and water ad libitum. All procedures involving animals were carried out in accordance with national guidelines for animal use in research and approved by Institutional Animal Care and Use Committee of San Raffaele Scientific Institute (Prot.N. 722/2016-PR D.lsg 26/2014 and Prot.N.842/2019-PR).

### Study design and experimental model

**Fig. 1:**
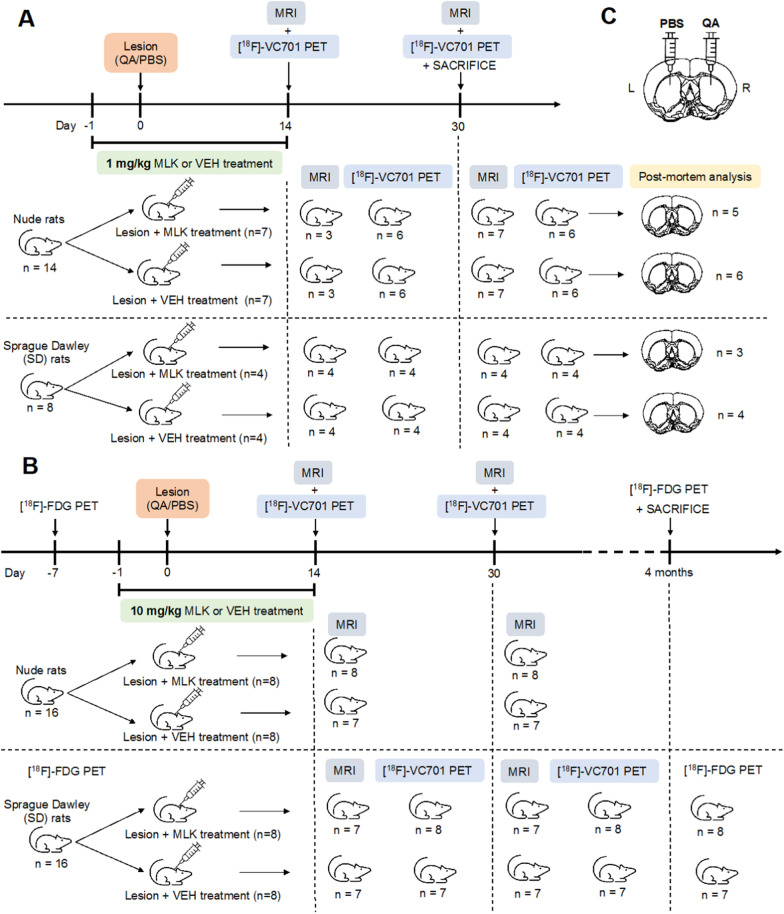
Experimental design and details about sample size for each group of rats (**A**, **B**). Representative illustration of the generation of the QA unilateral cytotoxic HD model (**C**)

Quinolinic acid (QA, Sigma-Aldrich) was used to induce striatal damage in all 54 rats. In brief, the lesion was generated by unilateral intracerebral injection of 210 nmol of QA in the right striatum using the following stereotaxic coordinates: AP, + 0.6; L, ± 2.8; V, 5.5 (Stoelting Europe, Dublin) [42] (see Additional file 1 for a representation of the coordinates used). An equivalent volume of phosphate-buffered saline (PBS) was injected in the contralateral striatum as a control. The whole procedure was carried out under general anesthesia (Isoflurane, 4% for induction and 2% for maintenance, in medical air; Isoflurane-Vet 100%, Merial Italy). Starting from the day before QA lesion, animals were treated intraperitoneally (i.p.) with montelukast (MLK; D.B.A. Italia s.r.l) or vehicle (VEH, i.e., saline solution) for 14 days (Fig. 1). Different groups of SD and nude rats received either a dose of 1 mg/kg (SD: *n* = 4 VEH and *n* = 4 MLK; nude: *n* = 7 VEH and *n* = 7 MLK) or 10 mg/kg (SD: *n* = 8 VEH and *n* = 8 MLK; nude: *n* = 8 VEH and *n* = 8 MLK). In both nude and SD rats, the protective effect of the 1 mg/kg dose was evaluated in vivo using MRI and [^18^F]-VC701 PET at 14 and 30 days post-injury and post-mortem by immunofluorescence (Fig. 1A). The effect of the 10 mg/kg dose in nude rats was evaluated only with MRI, due to the impossibility to access PET facilities during Covid-19 lockdown. SD rats that received 10 mg/kg were evaluated with both [^18^F]-VC701 PET and MRI at 14 and 30 days post-lesion and underwent also a 2-Deoxy-2-[^18^F]fluoroglucose ([^18^F]-FDG) PET before and at 4 months after QA (Fig. 1B), which was used to assess glucose metabolism and metabolic connectivity.

Sample size for [^18^F]-VC701 PET studies was calculated based on mean and standard deviation values from a previous [^18^F]-VC701 PET study in lesioned striatum at 7 days after intrastriatal QA injection [43]. We used G*Power Software (http://www.gpower.hhu.de/), setting 1-*β* = 0.8 and *α* = 0.05 with the hypothesis of a 20% of tracer uptake reduction in the lesioned striatum in MLK-treated animals, considering nude and SD rats separately. For [^18^F]-FDG PET studies, sample size was estimated based on a previous [^18^F]-FDG PET on QA-lesioned rats [44]. More details about the sample size for each experiment are reported in Fig. 1.

### Tissue collection, immunofluorescence and quantitative analysis

Nude and SD rats treated with the low MLK dose (1 mg/kg) and their controls were killed by perfusion at the end of MRI and [^18^F]-VC701 PET monitoring. Animals were deeply anesthetized with 1.7% tribromoethanol solution (20 µl/mg, i.p) and intracardially perfused with 0.1 M phosphate buffer (PB) (pH 7.4) followed by 4% paraformaldehyde (PFA) in 0.1 M PB. Brains were removed, post-fixed in PFA 4% for 24 h at 4 °C and then cryo-protected in 30% sucrose (Sigma-Aldrich) diluted in 0.1 M PB. Brains were embedded and frozen in cryostat medium (Killic, Bio-Optica, Milan, Italy) and 40 µm sections were cut on the cryostat (Leica) for histological analysis by immunofluorescence.

For immunofluorescence (IFL), brain sections containing the striatum were permeabilized with 0.5% Triton X-100 (Sigma-Aldrich) and blocked with 10% donkey serum (NDS) (Sigma-Aldrich) for 30 min at room temperature (RT). Here, we sought to assess neuroinflammatory markers, in particular activated microglia, marked by Iba1 and astrogliosis, marked by GFAP. In addition, we wanted to evaluate microglia phenotypes, by measuring the expression of mannose receptor (MannR), a marker of the anti-inflammatory phenotype and iNOS, a marker of the pro-inflammatory phenotype, as well as their co-localization with Iba1, GFAP and CD68, a macrophage marker, in the lesioned striatum. Thus, sections were incubated in 0.5% Triton X-100 with 2% NDS overnight at 4 °C with the following primary antibodies and dilutions: anti-Iba1 (rabbit, 1:1000; 019-19471; Fujifilm Wako Chemicals, U.S.A. Corporation, Richmond VA); anti-Iba1 (goat, 1:600; ab5076; Abcam; Cambridge, UK); anti-GFAP (rabbit, 1:500; Z0334; DAKO Cytomation, Agilent, Santa Clara, CA, USA); anti-iNOS (rabbit; 1:400; orb13614; Biorbyt, Cambridge, UK); anti-mannose receptor (rabbit; 1:500; ab64693; Abcam; Cambridge, UK); anti-CD68 (mouse; 1:500, MCA1957; Bio-Rad, Hercules, CA, USA). The following day, the sections were incubated for 2 h at RT with specific secondary antibodies (Cy^TM^3 AffiniPure anti-rabbit and anti-goat; Alexa Fluor anti-rabbit; Jackson Immunoresearch Laboratories, West Grove, PA, USA), and finally with DAPI (1:100) for 3 min, to stain the nuclei. Brain slices were then washed, mounted on 2% gelatin-coated slides and cover slipped with Mowiol embedding medium.

Images were acquired at Nikon Eclipse 90i (Nikon, Melville, NY) confocal microscope (1 µm z-step size, acquisition speed 100 Hz, format 1024 × 1024 pixels; 20× magnification for GFAP and Iba1 analysis, 40× magnification for iNOS and MAnnR analysis), examining 3 sections containing the lesioned striatum for every animal and acquiring at least 4 images for each section.

For the analysis of astrogliosis (GFAP) and microglial activation (Iba1), and iNOS and MannR-positive signals, the confocal images of positive immunolabelling were converted in black and white images, then the density of immunopositive pixels over the threshold in the field of view was quantified, for each experimental group, using ImageJ software (NIH).

### MRI data acquisition

All magnetic resonance imaging (MRI) studies were performed on a 7 T preclinical scanner (Bruker, BioSpec 70/30 USR, Paravision 6.0.1), equipped with 450/675 mT/m gradients (slew-rate: 3400–4500 T/m/s; rise-time 140 µs) and a circular polarized rat body volume coil with an inner diameter of 40 mm. All rats underwent imaging under inhalational anesthesia (Isoflurane, 3% for induction and 2% for maintenance in 2L/minute oxygen); lying prone on MRI bed and controlled with a dedicated temperature control apparatus to prevent hypothermia, having breathing rate and body temperature continuously monitored (SA Instruments, Inc., Stony Brook, NY, USA). MRI protocol included Rapid Acquisition with Relaxation Enhancement (RARE) T2-weighted sequence in coronal plane (slices 30, thickness 0.70 mm, gap = 0, TR/TE = 4000/48 ms, MTX = 180 × 180, FOV = 22 × 22 mm). Extraction of the lesion volume was performed manually using the Medical Image Processing, Analysis, and Visualization (MIPAV) software. Manual segmentation was performed by manually drawing a volume of interest (VOI) for the lesion (ipsilateral hemisphere, including edema), and one around both left and right total brain hemisphere (contralateral and ipsilateral) from the olfactory bulbs up to the cerebellar region. Corrected lesion volumes were obtained by normalizing to the ratio between healthy and lesioned hemispheres.

### PET data acquisition

#### [^18^F]-VC701 PET imaging

[^18^F]-VC701 PET was performed to assess neuroinflammation by applying the TSPO-specific radioligand [^18^F]-VC701, in line with previous work from our lab [43, 45]. PET images were acquired with the YAP(S)-PET II small animal tomograph (ISE S.r.l., Pisa, Italy) in the three-dimensional mode. Animals were injected in the tail vein with 6.51 ± 1.83 MBq of [^18^F]-VC701 tracer for SD rats and with 8.04 ± 1.5 MBq for nude rats. After 120 min of radiotracer uptake, animals were acquired for 30 min (6 frames of 5 min each) under gas anesthesia (2% isoflurane in medical air). Images were reconstructed using the expectation maximization (EM) algorithm, calibrated with dedicated phantom and corrected for radioisotope decay (T_1/2_ of ^18^F: 109.8 min). After that, PET images were manually co-registered to MRI scans. PMOD 4.1 software (Zurich, Switzerland) was used to calculate average radioactivity values in left and right striatum by applying the VOI brain template Px Rat-W. Schiffer [46]. Radiotracer uptake values for each VOI were expressed as percentage of injected dose per gram (%ID/g).

#### [^18^F]-FDG PET imaging

[^18^F]-FDG PET experiments were performed using an advanced PET/CT scanner ($\beta$-cube®/X-cube®, Molecubes, Gent, Belgium) which exhibits higher resolution and sensitivity properties (field of view: 130 mm × 72 mm, spatial resolution: 0.85 mm, sensitivity: 12.6%) than the tomograph used for [^18^F]-VC701 PET. [^18^F]-FDG, a radiotracer that measures glucose metabolism, was prepared as for clinical use (European Pharmacopoeia VIII Edition). Animals were food-deprived for 12 h prior to the experiment and injected in the tail vein with an average of 10.7 ± 0.5 MBq of [^18^F]-FDG for SD rats and with 15.54 ± 0.73 MBq for nude rats. After 40 min of uptake, PET images were acquired for 20 min under gas anesthesia (2% isoflurane in medical air). During acquisition, animals were constantly monitored for respiratory rate and maintained at constant temperature with a heating pad. [^18^F]-FDG PET images were reconstructed (GPU-based iterative OSEM reconstruction; 30 iterations, 400 µm), automatically corrected for radioisotope decay and manually co-registered to MRI. Images were then quantified with PMOD software by applying a VOIs template based on the Schiffer rat brain atlas [46] and extracting the mean tracer uptake values from the following 28 brain areas: left and right olfactory bulb (L_OlfB, R_OlfB), left and right prefrontal cortex (L_PFC, R_PFC), left and right frontal cortex (L_Front, R_Front), left and right orbitofrontal cortex (L_OFC, R_OFC), left and right cingulate cortex (L_Cg, R_Cg), left and right motor cortex (L_MC, R_MC), left and right somatosensory cortex (L_SSC, R_SSC), left and right striatum (L_S, R_S), left and right anterior hippocampus (L_AntHip, R_AntHip), left and right posterior hippocampus (L_PostHip, R_PostHip), left and right thalamus (L_Tha, R_Tha), left and right hypothalamus (L_HTha, R_HTha), left and right midbrain (L_MB, R_MB), left and right cerebellum (L_CRB, R_CRB). These brain regions were selected from the atlas since they are known to be directly or indirectly involved in HD pathogenesis [47]. Radiotracer uptake values were expressed as %ID/g and normalized dividing them by the mean whole-brain uptake in order to account for intra-subject variability in glucose metabolism.

### Statistical analysis

Statistical analyses were performed using Prism 8 (GraphPad Software Inc., CA, USA) and R software (R Core Team). In particular, a mixed-effects two-way ANOVA and ordinary one-way ANOVA with bootstrap re-sampling, followed by Sidak’s multiple comparison correction, were employed. Statistical significance was accepted when *p* < 0.05.

[^18^F]-FDG PET regional values were also analyzed by applying a covariance analysis method to assess metabolic connectivity between brain areas. This analysis is based on the observation that regions with significantly correlated [^18^F]-FDG uptake values are also functionally connected [48, 49]. The metabolic connectivity analysis was performed by using the brainGraph toolbox (https://github.com/cwatson/brainGraph) available in R. In detail, Spearman’s correlation coefficient (ρ) was calculated for each pair of brain regions and a 28 × 28 weighted undirected correlation matrix was obtained for each treatment group (pre-lesion VEH, pre-lesion MLK, QA + VEH and QA + MLK) where each interregional correlation coefficient (*ρ*) indicates the strength of connection. The obtained ρ values were then converted into z-scores by applying Fisher transformation, with the following equation:

$$z= \frac{1}{2}\mathrm{ln} \frac{(1+)}{(1-)}.$$

To statistically compare each correlation coefficient between different treatment groups, we performed a Z test, using the following equation:

$$Z= \frac{{z}_{1}-{z}_{2}}{\sqrt{\frac{1}{{n}_{1}-3}+\frac{1}{{n}_{2}-3}}},$$

where ${z}_{1}$ and ${z}_{2}$ represent the z-scores from the two groups compared and ${n}_{1}$ and ${n}_{2}$ the sample size. The associated p-values were obtained from Z test results to determine the significance of between-group differences and false discovery rate (FDR) was applied to correct the p-values for the total number of tests.

Finally, a network analysis was performed on the same data to characterize the topology of the patterns of metabolic connectivity and each node regional properties. In particular, we focused on measures of centrality, such as node degree and betweenness centrality, but also measures of functional integration, such as local efficiency. Node degree is the number of edges (i.e., connections between nodes) connected to a given node (i.e., VOIs), while betweenness centrality is defined as the fraction of all shortest paths in the network passing through a node [50]. Both these measures are important to identify central nodes (hubs) that are connected to several other nodes [50]. On the other hand, functional integration can be explained as the efficiency of a network to combine information from different regions and can be measured by local efficiency [50]. A permutation test was applied to determine between-group statistical differences in these network metrics.

## Results

### Evaluation of the neuroprotective effects of MLK treatment on MRI lesion volume

**Fig. 2:**
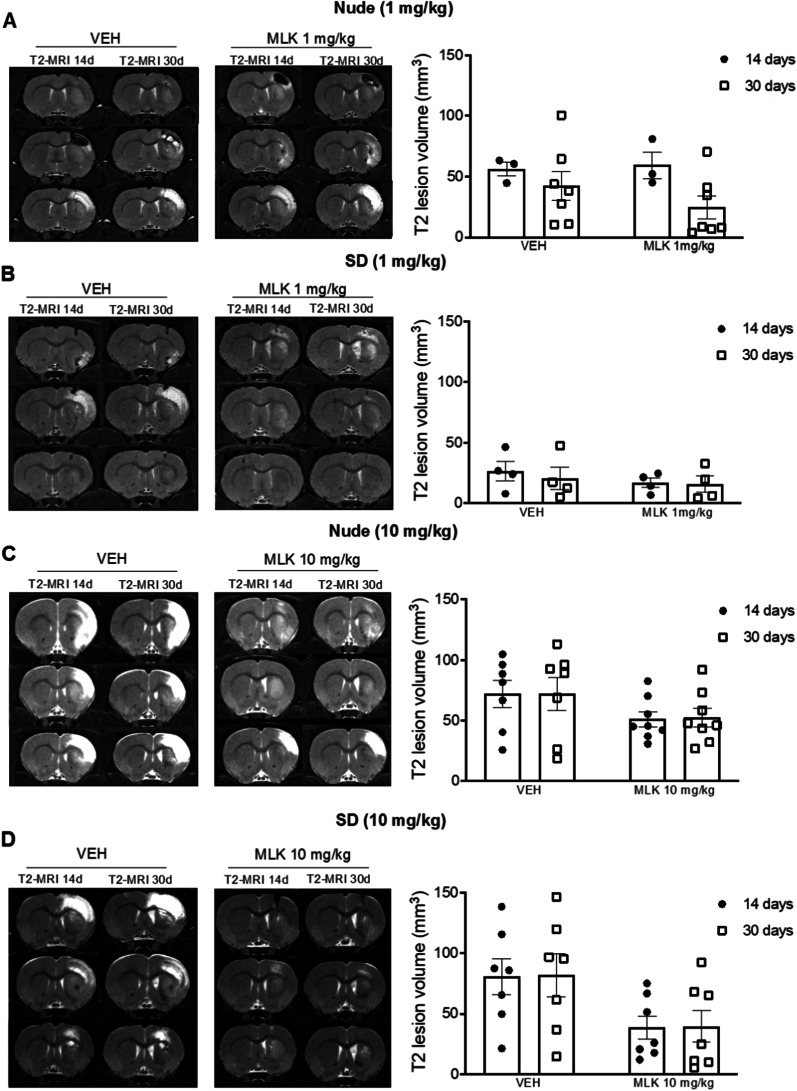
Representative T2-weighted MRI coronal images showing QA striatal lesion. Lesion volume was evaluated at 14 and 30 days after QA administration in nude and SD rats treated with 1 mg/kg (**A**, **B**) or 10 mg/kg (**C**, **D**) of MLK, in comparison with vehicles. Data are plotted as mean ± standard error of the mean (SEM). Mixed-effects two-way ANOVA with bootstrap

MR imaging study was conducted to assess potential structural modifications induced by MLK treatment on QA brain injury. In the lesioned hemisphere, QA administration induced an enhancement of T2-MRI signal in the striatum and in some rats this effect was present also in ipsilateral cortical regions, especially near the striatum (Fig. 2). We have previously reported similar structural alterations in the same model in an independent study [51]. We also observed ventricle enlargement in most animals, which is a recurrent feature in HD patients [52] (Fig. 2). A mixed-effects two-way ANOVA with bootstrap revealed no significant interaction between time point (within-subjects) and treatment (between-subjects) (MLK 1 mg/kg) or significant main effects of time or treatment on MRI lesion volume, in both nude and SD rats (Fig. 2A, B). The same results were obtained in rats treated with an higher MLK dose (10 mg/kg) (Fig. 2C, D); however, we note that here the main effect of treatment approached significance (*F*_1,12_ = 4.533, *p* = 0.06), suggesting a reduction in lesion volume in SD rats when MLK-treated were compared with VEH-treated animals (Fig. 2D).

### Evaluation of the neuroprotective effects of MLK treatment on neuroinflammation

**Fig. 3:**
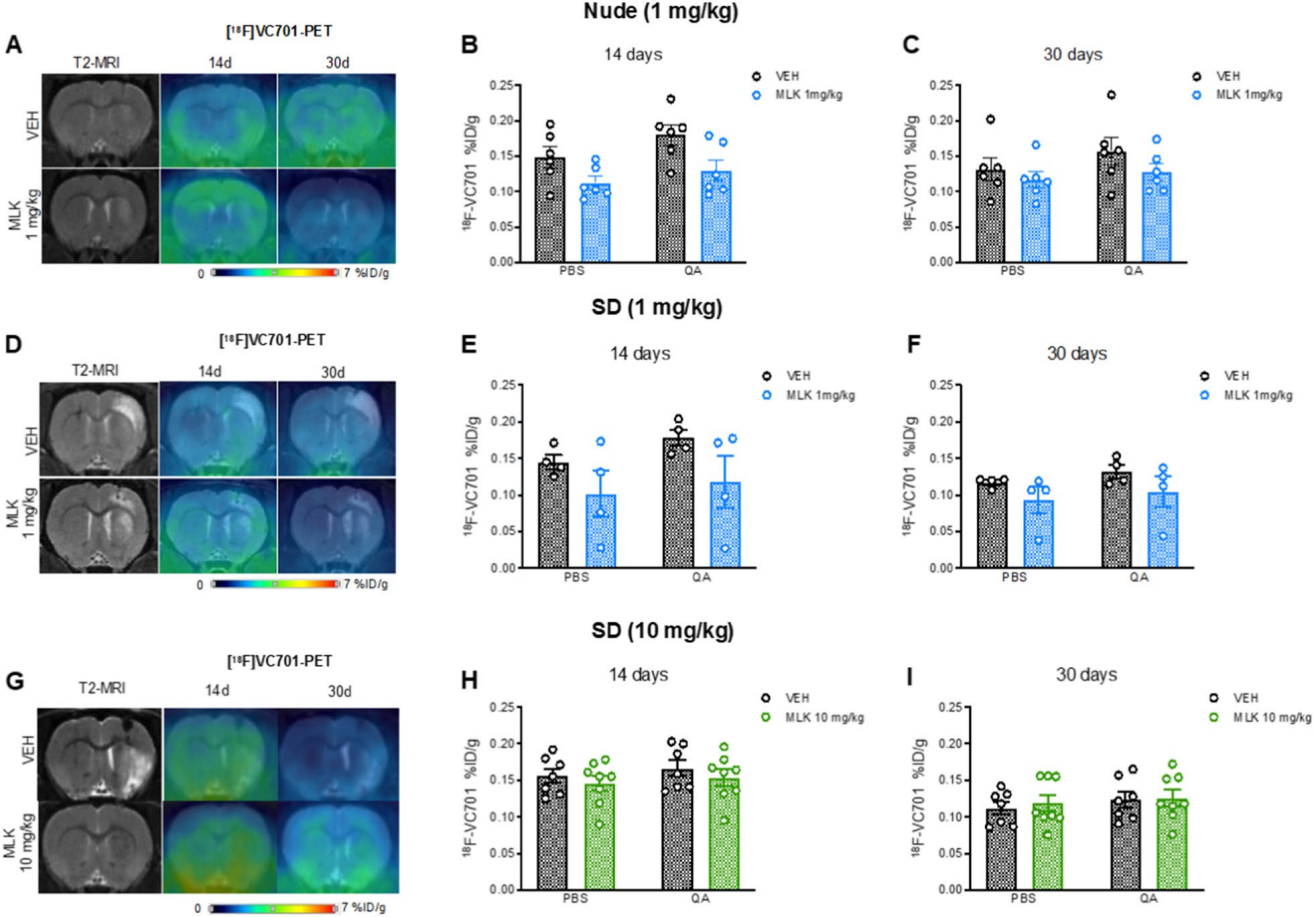
Representative [^18^F]-VC701 PET coronal images of nude (**A**) and SD rats (**D**, **G**) injected with QA and treated with VEH or MLK, co-registered to MRI scans from the same time points. Striatal [^18^F]-VC701 uptake was evaluated at 14 (**B**, **E**, **H**) and 30 days (**C**, **F**, **I**) post-lesion both in the left PBS-injected and the right QA-injected striata and it is expressed as %ID/g. Data are plotted as mean ± SEM. Mixed-effects two-way ANOVA with bootstrap

In the low MLK dose (1 mg/kg) group, a mixed-effects ANOVA with bootstrap revealed no significant interaction between lesion hemisphere (within-subjects) and treatment (between-subjects) in nude rats, at both time points (Fig. 3B, C). QA lesion had a significant main effect on [^18^F]-VC701 uptake both at 14 (*F*_1,10_ = 35.77, *p* < 0.0001; Fig. 3B) and 30 days after lesion (*F*_1,10_ = 29.13, *p* < 0.0001; Fig. 3C), suggesting an increase in neuroinflammation. At 14 days, we also observed a significant main effect of treatment (*F*_1,10_ = 5.470, *p* = 0.048; Fig. 3B), driven by decreases in [^18^F]-VC701 uptake in the group treated with MLK irrespective of the lesion hemisphere. We did not find any significant interaction between lesion hemisphere and treatment in immunocompetent SD rats (Fig. 3E, F). A significant main effect of QA lesion on [^18^F]-VC701 uptake was detected also in SD rats at 14 (*F*_1,6_ = 24.39, *p* = 0.02; Fig. 3E) and 30 days post-QA (*F*_1,6_ = 9.265, *p* = 0.02; Fig. 3F), but the main effect of treatment was not significant.

Similarly to what we observed in the group of animals treated with MLK 1 mg/kg, we also did not observe an interaction between lesion hemisphere and treatment in animals treated with MLK 10 mg/kg (Fig. 3H,I). QA lesion had a significant effect, increasing [^18^F]-VC701 uptake in the lesioned striatum compared to the non-lesioned one in SD rats at both 14 days (*F*_1,13_ = 15.68, *p* = 0.019; Fig. 3H) and 30 days after lesion (*F*_1,13_ = 10.47, *p* = 0.009; Fig. 3I); however, we did not observe any significant main effect of treatment.

**Fig. 4:**
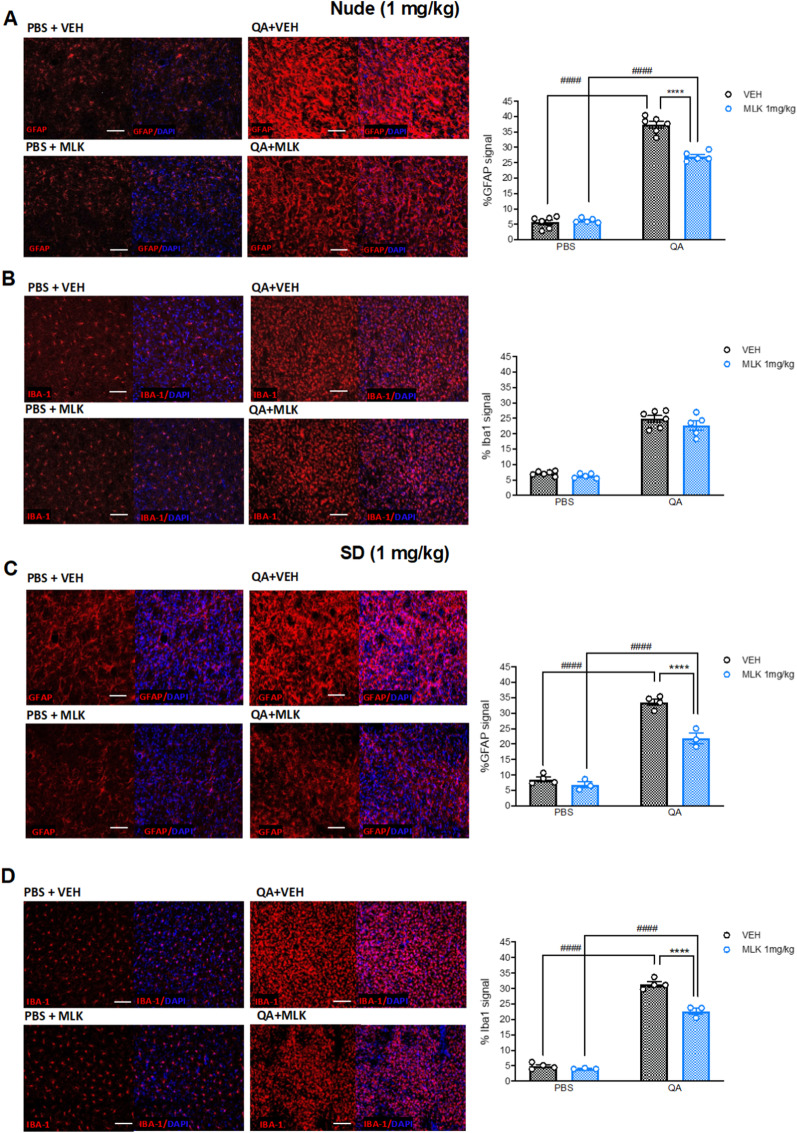
Post-mortem immunofluorescence analysis of striatal neuroinflammation in nude and SD rats at 30 days post-lesion. GFAP- and Iba1-positive signals were measured in the PBS- (left panels) and QA-injected striatum (right panels) of nude (**A**, **B**) and SD rats (**C**, **D**) after treatment with VEH (top panels, black histogram bars) or 1 mg/kg of MLK (bottom panels, blue histogram bars). Mixed-effects two-way ANOVA with bootstrap: ****p < 0.0001, VEH vs MLK; ^####^*p* < 0.0001, PBS vs QA. Scale bars: 100 µm

Since the target of [^18^F]-VC701, TSPO, is expressed by both microglia and astrocytes, we performed IFL on post-mortem brain tissue from the animals treated with 1 mg/kg of MLK to measure Iba1- and GFAP-positive signals (that, respectively, identify activated microglia and astrocytes) in the striatum. A mixed-effects two-way ANOVA with bootstrap accounting for the effects of lesion hemisphere (within-subjects) and treatment (between-subjects) on GFAP and Iba1 signal intensity in nude rats revealed an interaction between the two factors for GFAP (*F*_1,9_ = 68.63, *p* < 0.0001; Fig. 4A) but not for Iba1 (Fig. 4B). As expected, after multiple comparison correction, we found that QA injection significantly increased GFAP-positive signal in comparison with the non-lesioned striatum in both VEH- (*p* < 0.0001) and MLK-treated animals (*p* < 0.0001). Treatment with 1 mg/kg of MLK significantly reduced GFAP-positive signal (*p* < 0.0001) in the lesioned striatum in comparison with VEH-treated rats. We only found a significant main effect of the lesion (*F*_1,9_ = 243.7, *p* < 0.0001) on Iba1 signal, but no significant effect of the treatment. In SD rats, mixed-effects two-way ANOVA with bootstrap revealed an interaction between the two factors (lesion × treatment) in both GFAP (*F*_1,5_ = 19.31, *p* = 0.007; Fig. 4C) and Iba1 (*F*_1,5_ = 29, *p* = 0.04; Fig. 4D). Post hoc analysis revealed a significant increase in GFAP-positive signal in the QA-lesioned striatum compared to the control one in both VEH- (*p* < 0.0001) and MLK-treated animals (*p* < 0.0001). This increase was diminished by MLK, which significantly reduced GFAP signal in the lesioned striatum compared with vehicles (*p* < 0.0001). Similar results were obtained for Iba1 signal: we observed a significant increase in the lesioned striatum compared to the healthy counterpart in VEH- (*p* < 0.0001) and MLK-treated rats (*p* < 0.0001), as well as a significant reduction in the lesioned striatum of MLK-treated animals in comparison with the vehicles (*p* < 0.0001).

**Fig. 5:**
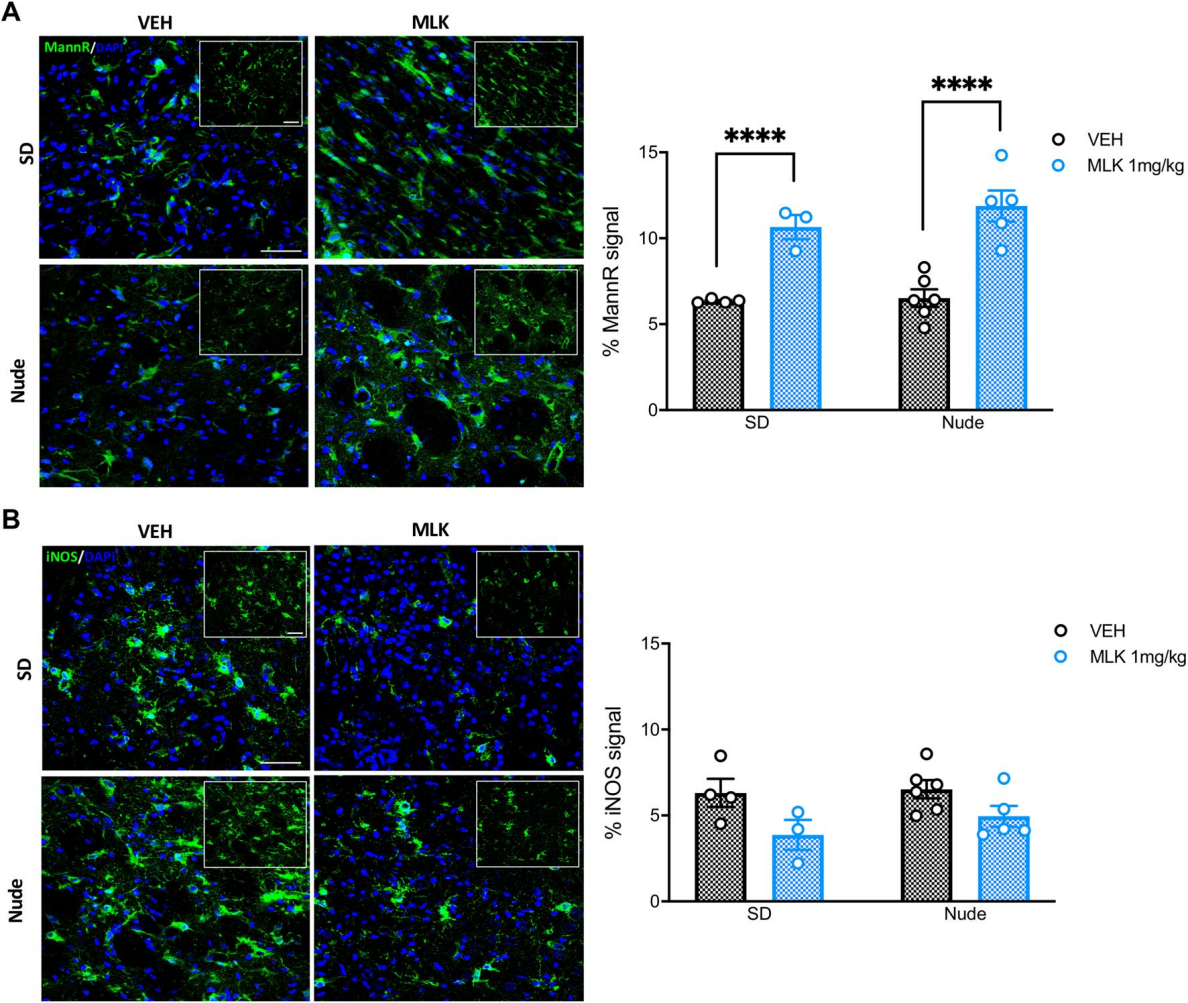
Post-mortem immunofluorescence analysis of striatal MannR (**A**) and iNOS expression (**B**) in the QA-lesioned striatum of SD (top panels) and nude rats (bottom panels) treated with VEH (left panels and black histogram bars) or 1 mg/kg of MLK (right panels and blue histogram bars) at 30 days post-lesion. One-way ANOVA with bootstrap: ****p < 0.0001, VEH vs MLK. Scale bars: 50 µm. Data are plotted as mean ± SEM

To better understand the nature of these effects, we also quantified the expression of MannR and iNOS signals, markers of anti-inflammatory and pro-inflammatory microglial phenotypes, respectively, as well as their co-localization with macrophages (CD68), astrocytes (GFAP) and microglia (Iba1), in the QA-lesioned striatum only (Fig. 5). One-way ANOVA with bootstrap revealed that MannR-positive signal intensity was significantly increased in the lesioned striatum of MLK-treated rats when compared to VEH-treated animals (*F*_3,14_ = 19.15, *p* < 0.0001), for both nude (*p* < 0.0001) and SD strain (*p* < 0.0001) animals (Fig. 5A). On the other hand, iNOS expression intensity in the lesioned striatum of MLK-treated rats showed a trend towards reduction compared with VEH-treated animals (*F*_3,14_ = 3.007, *p* = 0.0659; Fig. 5B). In addition, we found that iNOS and MannR did not co-localize with CD68- and Iba1-positive cells (Additional file 2: Fig. S2A, B, D, E), but co-localized with GFAP-expressing cells (Additional file 2: Fig. S2C, F).

### Evaluation of the neuroprotective effects of MLK treatment on glucose metabolism

**Fig. 6:**
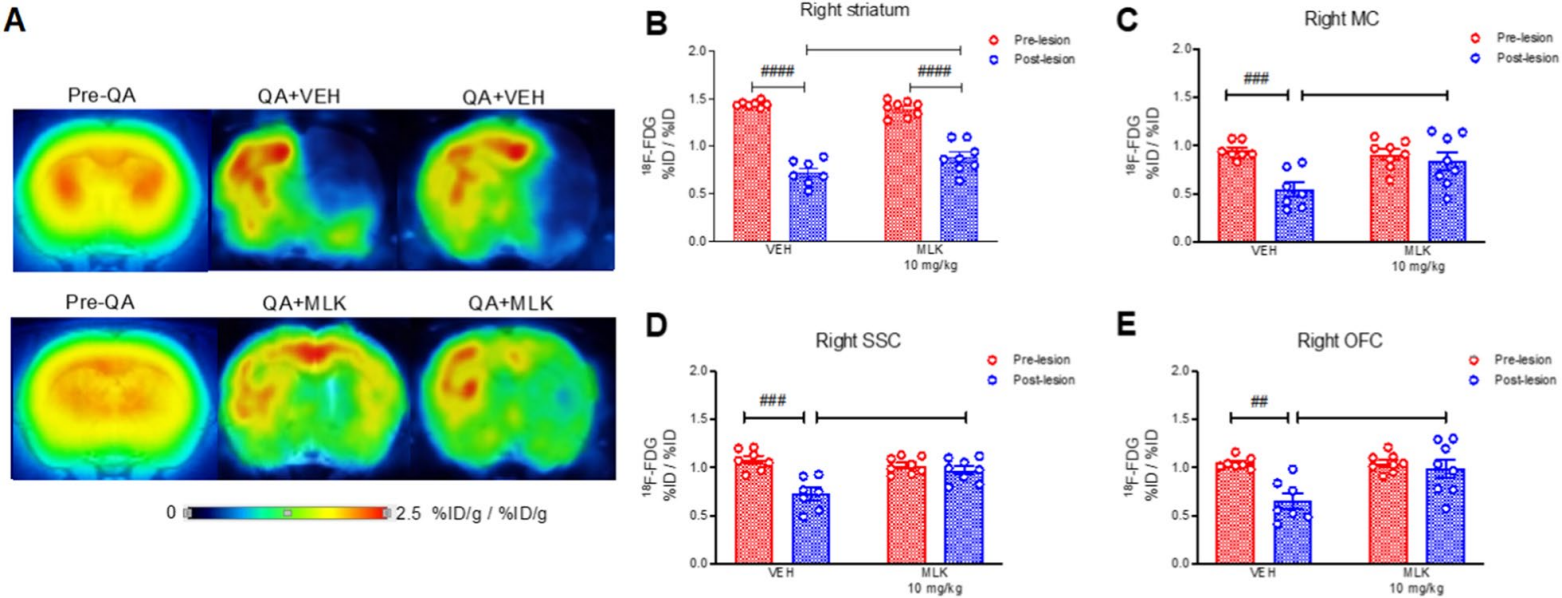
Representative [^18^F]-FDG PET coronal images of SD rats before (red histogram bars) and 4 months after QA injection (blue histogram bars), treated with VEH or 10 mg/kg of MLK, co-registered to MRI (**A**). [^18^F]-FDG uptake was evaluated in the right striatum (**B**) and cortical regions (**C**–**E**) and it is expressed as a ratio to the global. Mixed-effects two-way ANOVA with bootstrap: ^##^*p* < 0.01, ^###^*p* < 0.001, ^####^*p* < 0.0001, pre-lesion vs post-lesion; *p < 0.05, **p < 0.01, VEH vs MLK. Data are plotted as mean ± SEM

SD animals used for the evaluation of the high dose of MLK (10 mg/kg) were monitored with [^18^F]-FDG PET before and 4 months after QA administration. At 4 months, the lesion was still evident (Fig. 6A) and we found a significant reduction in [^18^F]-FDG uptake in the regions of the lesioned hemisphere of VEH-treated rats compared with the baseline. In particular, we focused on the right striatum, where the lesion was first induced, and some cortical regions of the lesioned hemisphere, such as the right MC, right SSC and right OFC (Fig. 6B–E). Using a mixed-effects two-way ANOVA with bootstrap, we found a significant interaction between QA lesion (within-subjects) and treatment (between-subjects) on [^18^F]-FDG uptake in all the above-mentioned VOIs (right striatum: *F*_1,13_ = 5.813, *p* = 0.034; right motor cortex: *F*_1,13_ = 7.987, *p* = 0.015; right somatosensory cortex: *F*_1,13_ = 12.02, *p* = 0.002; right orbitofrontal cortex: *F*_1,13_ = 8.214, *p* = 0.02). Furthermore, post hoc analysis showed a significant reduction in [^18^F]-FDG uptake after lesion in the right striatum, in both VEH- (*p* < 0.0001) and MLK-treated animals (*p* < 0.0001). However, MLK treatment was able to attenuate this reduction in lesioned animals (*p* = 0.0208) (Fig. 6B). Regarding cortical regions of the ipsilesional hemisphere, we observed a significant reduction in radiotracer uptake in the right MC of VEH-treated rats after QA lesion, compared to pre-lesion (*p* = 0.0008); this reduction was attenuated by MLK treatment, as shown by the higher values of [^18^F]-FDG uptake compared to vehicles (*p* = 0.0106) (Fig. 6C). Similar results were obtained also for the right SSC (Fig. 6D) and right OFC (Fig. 6E), where we observed a significant reduction in [^18^F]-FDG uptake in VEH-treated animals that received QA compared to pre-lesion (right SSC: *p* = 0.0002; right OFC: *p* = 0.001). In both regions, the administration of 10 mg/kg of MLK prevented this reduction, with [^18^F]-FDG uptake values in QA + MLK rats higher than those measured in QA + VEH rats (right SSC: *p* = 0.0013; right OFC: *p* = 0.0026).

### Effects of QA-induced lesion and MLK treatment on brain interregional metabolic connectivity

**Fig. 7:**
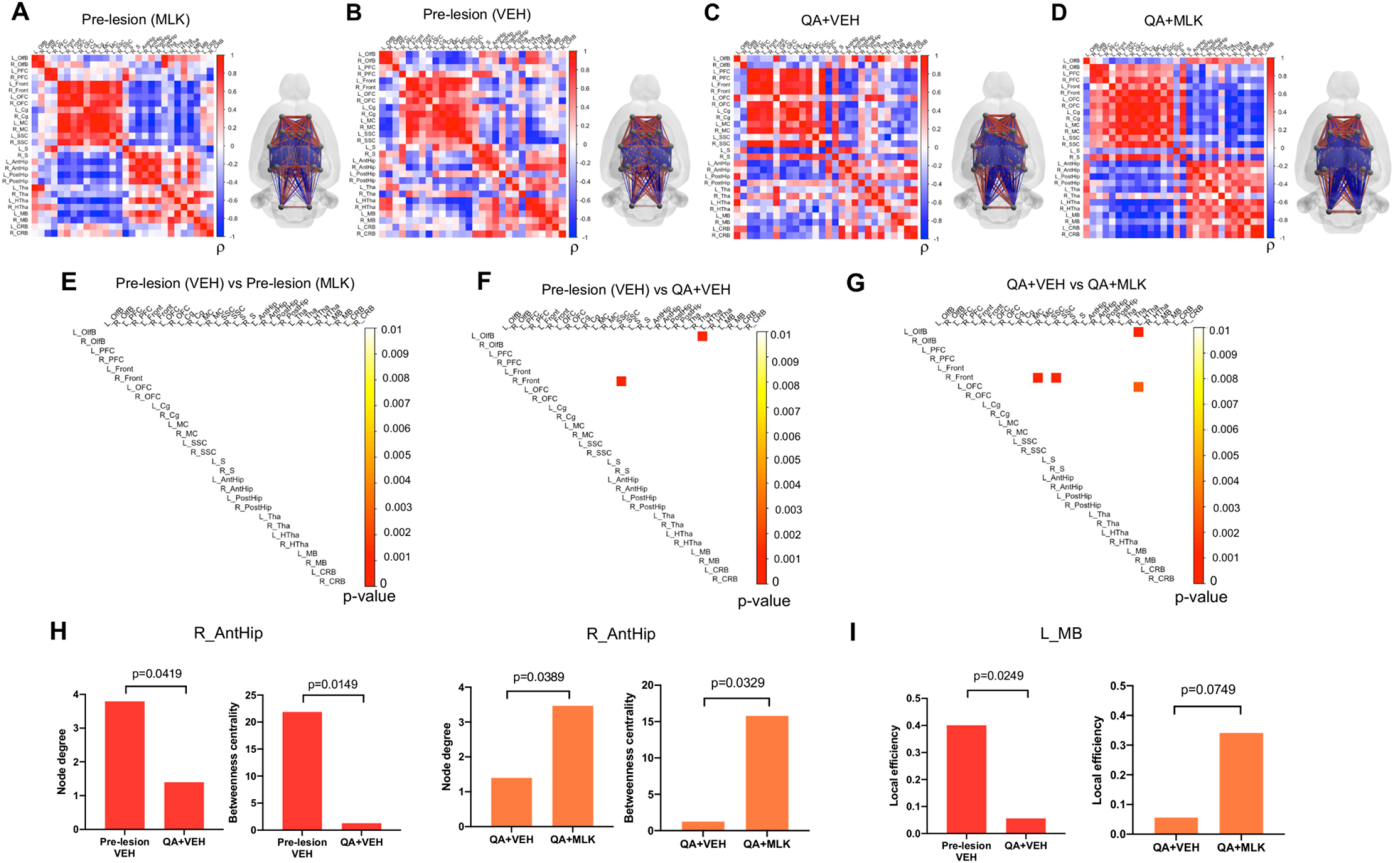
[^18^F]-FDG PET correlation matrices of pre-lesion (**A**, **B**), QA + VEH (**C**) and QA + MLK rats (**D**) and their respective brain network representation (on the right of each matrix), where red edges indicate positive and blue edges negative correlations between nodes (in grey) and the size of each edge is proportional to the size of the correlation coefficient. Correlation matrices are statistically compared between groups and only the connections that are significantly different between the two groups (*p* < 0.01 with FDR correction) are showed in the upper triangular matrix (**E**–**G**). Node degree, betweenness centrality and local efficiency are statistically compared between groups using permutation test (**H**, **I**). All brain networks are visualized using BrainNet Viewer [53]

In Fig. 7, we present [^18^F]-FDG PET correlation matrices and connectivity graphs, one for each group of SD rats.

In the baseline condition (Fig. 7A, B), we observed a pattern of positive correlations between ipsi- and contra-lesional cortical regions (upper left part of the matrices) and between ipsi- and contra-lesional sub-cortical regions (lower right part of the matrices). On the contrary, cortical and subcortical regions generally correlated negatively (upper right and lower left parts of the matrices). As expected, the baseline matrices from the two treatment groups, those treated with VEH and those treated with MLK, did not statistically differ from each other (Fig. 7E). From the comparison between pre- and post-lesion in the VEH group we observed that the administration of QA caused a reorganization of interregional correlations, compared to the baseline (Fig. 7B, C). Overall, QA injection increased intra- and inter-hemisphere correlation between cortical regions. Cortical regions were positively correlated with the lesioned striatum and negatively correlated with the contralesional one. After FDR correction, we found that the correlation between the ipsilesional frontal cortex and SSC was significantly higher after lesion (*p* < 0.0001; Fig. 7F). The same finding was observed for the correlation between contralesional olfactory bulb and hypothalamus (*p* < 0.0001; Fig. 7F). MLK treatment partially modified regional connectivity to pre-lesion conditions, at both cortical and subcortical levels. In particular, MLK decreased the correlation between ipsilesional frontal cortex and SSC in comparison with QA + VEH animals (*p* < 0.0001; Fig. 7G). Similar effects were observed between contralesional olfactory bulb and hypothalamus and between ipsilesional frontal and motor cortex, where we found a reduced correlation in QA + MLK compared to QA + VEH rats (*p* < 0.0001; Fig. 7G). Finally, the correlation between ipsilesional hypothalamus and OFC was significantly different between the two groups (*p* = 0.0027): it was positive in the QA + VEH group and returned negative in the QA + MLK group, as it was in the baseline condition (Fig. 7G).

The network analysis revealed a significant decrease in node degree (*p* = 0.0419) and betweenness centrality (*p* = 0.0149) in the ipsilesional anterior hippocampus of QA + VEH animals, compared to pre-lesion (Fig. 7H). Interestingly, MLK treatment counteracted this reduction by significantly increasing node degree (*p* = 0.0389) and betweenness centrality (*p* = 0.0329) in the ipsilesional anterior hippocampus as compared to VEH-treated rats, to levels close to the baseline (Fig. 7H). Furthermore, in the contralesional midbrain we observed a significant decrease in local efficiency (*p* = 0.0249) in the QA + VEH group compared to baseline, while we observed a trend for increase (*p* = 0.0749) in QA + MLK rats when compared to the QA + VEH group (Fig. 7I).

## Discussion

In this work, we sought to explore the neuroprotective effects of MLK treatment on QA-induced neurotoxicity in rats, by evaluating tissue damage and metabolism, neuroinflammation and brain metabolic connectivity. We studied both immunocompetent SD and athymic nude rats to investigate the contribution of T-cells to the neuroinflammatory response to the lesion and potential effects of MLK. Results from in vivo imaging studies revealed a similar susceptibility of both strains to MLK administration and neuroinflammatory response to QA as reported by post-mortem immunofluorescence data. Overall, MLK exhibited a significant neuroprotective effect by reducing astrogliosis, preserving regional brain metabolism and metabolic connectivity from QA-induced insult in cortical and subcortical regions.

As expected, QA administration enhanced T2-weighted signal in the right striatum and in some cortical regions (for some of the animals), replicating previous studies [51]. However, MLK failed to attenuate the volume of QA-induced lesions, as we only found a non-significant trend for decreased lesion volume in animals treated with the 10 mg/kg dose, which was more evident in SD rats. Similar null findings were obtained in the [^18^F]-VC701 PET studies, where MLK treatment did not attenuate the increase of TSPO signal induced by QA injection. However, we cannot exclude that our sample size might have lacked power to detect smaller treatment effects with [^18^F]-VC701 PET imaging.

TSPO-specific radiopharmaceuticals are largely used, in both preclinical and clinical studies, to image microglia/macrophage activation and to a lesser extent astrocytosis [54, 55]. In order to better understand the nature of TSPO signal and overcome the sensitivity limits of PET imaging, we also measured the striatal expression of Iba1 and GFAP*,* markers of microglia and astrocyte activation, respectively, in post-mortem rat brain slices. At 30 days after QA lesion, the administration of 1 mg/kg of MLK produced an attenuation of the increase in Iba1 in the lesioned striatum of SD rats—this effect was not present for nude rats. For both strains, we found that MLK attenuated increases in GFAP signal intensity; however, MLK did not reduce GFAP to the levels observed in the contralateral non-lesioned striatum, as a result of the inflammatory state persisting at the striatal level 30 days after QA injection [35].

Microglia, as peripheral macrophages, can acquire different phenotypes depending on the brain microenvironment. These phenotypes can be simplified as M1 or M2 phenotypes, mainly associated with a pro-inflammatory or an anti-inflammatory activity, respectively [56, 57]. Therefore, to obtain a clearer picture of the effect of MLK on brain immune cells, we also looked at the expression of iNOS, that preferentially labels M1 phenotype, and MannR, marker of M2 phenotype [58–60]. We found a significant increase in MannR as well as a trend towards reduction in iNOS signal in the lesioned striatum of MLK—compared to VEH-treated rats, in both strains. However, surprisingly, MannR and iNOS signals mainly co-localized with GFAP-expressing cells, suggesting that changes in expression of these markers are likely to be driven by astrocytes. While this observation might be counterintuitive, we note that the expression of MannR and iNOS in GFAP-positive cells has been described in the literature [60–64], even if their role in astrocyte regulation is not fully understood yet. MannR is known to be upregulated following anti-inflammatory stimuli, while it is downregulated by pro-inflammatory cytokines or immune stimuli [59, 62]. After induction of unilateral focal cortical ischemia in the rat brain, MannR was found to be highly expressed in GFAP-positive cells in the lesioned side, but not in the non-lesioned one [61]. The authors suggested that MannR, besides its functions in innate immunity, might be involved in regenerative processes induced by acute brain injury. Taken together, our results suggest that MLK treatment might have an anti-inflammatory effect at 30 days after QA lesion, but this effect is likely to be primarily associated with astrocytic cells. In line with our results, previous in vitro findings revealed that MLK attenuates the proliferation of primary cultured rat astrocytes in response to metabolic insults (oxygen–glucose deprivation) in a CysLT1R-mediated manner [65]. Interestingly, NMDA injection has been shown to upregulate CysLT1R expression in the brain [66]. Since high concentrations of QA also stimulate the NMDA receptor, it is possible that CysLT1R might be involved in QA-induced excitotoxicity and that blocking CysLT1R engagement using MLK might have a beneficial effect.

MLK antagonizes not only CysLT1R, but also the GPR17 receptor [27]. CysLT1R is predominantly expressed in astrocytes, microglia and at lower levels also in oligodendroglia. The GPR17 receptor is mainly expressed in cells of neuronal lineage and oligodendrocytes, where it regulates neurogenesis and myelination [27, 67]. Therefore, beyond neuroinflammation, it is not implausible that MLK might similarly have effects on neuronal function and plasticity. Based on this hypothesis, we evaluated long-term effects of MLK on regional brain function using [^18^F]-FDG PET imaging. [^18^F]-FDG is a well-established brain marker of synaptic function used in both clinical and preclinical studies. Importantly, [^18^F]-FDG uptake has been found to be significantly decreased in caudate, putamen and in the frontal lobe of HD patients and uptake in these regions correlated with disease severity [68]. Accordingly, we found that QA administration led to a reduction in [^18^F]-FDG uptake in the striatum, but also in several other cortical regions connected with the striatum, at 4 months after QA injection. MLK produced a significant protective effect on metabolism, preserving regional glucose consumption levels in ipsilesional cortical areas, such as the right MC, SSC and OFC; however, the same effect was not observed in the lesioned striatum, where MLK only produced attenuation of the decreases in metabolism, but not a normalization. Taken together, these data indicate that MLK treatment exerted protective effects on brain metabolism, preventing extension of QA-induced excitotoxicity to the cortex and attenuating intrastriatal effects of QA.

Regional brain [^18^F]-FDG retention is complex and involves not only neurons, but also astrocytes [69]. Moreover, it is well known that at sub-nanomolar concentrations QA is toxic for both neurons and astrocytes [70], therefore modifications observed in regional glucose metabolism levels might be associated with both cell types. Given our results on the effect of MLK in reducing QA-induced astrogliosis, we cannot exclude the involvement of these cells in the protective action exerted by MLK on regional metabolism. Region-specific metabolic reprogramming of astrocytes in HD preclinical models has been recently proposed by Polyzos et al*.* [71]; thus, a possible effect of MLK on astrocyte metabolism warrants further investigations.

[^18^F]-FDG PET data can be also useful to explore how different brain regions are functionally interconnected between each other. Abnormalities in metabolic connectivity have been previously reported in preclinical models of neurodegenerative disorders and stroke [72, 73]. Here, we found that the injection of QA generally resulted in an increase in metabolic connectivity for both lesioned and non-lesioned hemispheres as compared to baseline, particularly in cortical and subcortical regions integrating the basal ganglia functional circuit. MLK treatment seems to prevent this effect, since MLK lesioned animals tend to show connectivity patterns that are more alike their respective baseline. We also applied a graph-based analysis on the same data in order to explore the effects of QA and MLK administration on metabolic network topology and found that QA lesion produced decreases in measures of centrality (both node degree and betweenness centrality) of the ipsilesional anterior hippocampus in the VEH group, which were counteracted by MLK treatment. On the other hand, in the contralesional midbrain we observed a significant reduction in local efficiency after lesion in the VEH group, which was counteracted by MLK treatment. These results highlight an important role of MLK in preserving the key role of hippocampus and midbrain as central nodes transmitting information within networks of the brain. These findings are compelling since both regions are known to be part of the brain networks controlling, for instance, grooming behavior or spatial learning, which have been described to be progressively impaired in the QA rat model of HD [74, 75].

Overall, using the QA injection model of the HD brain pathology, we showed that MLK can exert neuroprotective effects by reducing astrogliosis, preserving regional brain metabolism and promoting plastic changes in metabolic connectivity across cortical and subcortical regions to bypass lesion-induced functional modifications. The QA injection model recapitulates most of the behavioral and pathological features of HD, but induces a strong and fast neurotoxic effect on striatal medium spiny neurons, unlike HD patients where progressive neuronal death has been described. Therefore, the recent development of novel preclinical models of HD will allow a better evaluation of MLK effects also in prodromic or early stages of the disease [76].

## Conclusions

In conclusion, our study lends support to the idea that MLK might merit further clinical investigation as a neuroprotective agent in HD patients and non-symptomatic carriers.

## Supplementary Information

**Additional file 1: Fig. S1.** Coronal section from the Rat Brain Atlas (Paxinos & Watson) [42] showing the stereotaxic coordinates used for intracerebral administration of QA, leading to the caudate putamen (CPu) as indicated by the red dot.

**Additional file 2: Fig. S2.** Representative confocal images from immunofluorescence co-localization analysis in the QA-lesioned striatum of nude and SD rats treated with either VEH or MLK 1 mg/kg, at 30 days post-lesion. Co-localization of CD68 and DAPI with MannR is represented in panel A and with iNOS in panel D. Co-localization of Iba1 and DAPI with MannR is represented in panel B and with iNOS in panel E. Finally, co-localization of GFAP and DAPI with MannR is represented in panel C and with iNOS in panel F. Scale bars: 50 µm.

## Data Availability

The raw data supporting the results of this study will be made available upon request.

## Abbreviations

HD: Huntington's disease
polyQ: Polyglutamine
HTT: Huntingtin
PET: Positron emission tomography
[^18^F]-FDG: 2-Deoxy-2-[^18^F]fluoroglucose
TSPO: Translocator protein
MLK: Montelukast
CysLT1R: Cysteinyl leukotriene receptor 1
GPR17: G protein-coupled receptor 17
CNS: Central nervous system
CysLTRs: Cysteinyl leukotriene receptors
BBB: Blood–brain barrier
QA: Quinolinic acid
NMDAR: NMDA receptor
MRI: Magnetic resonance imaging
SD: Sprague Dawley
i.p.: Intraperitoneal
VEH: Vehicle
IFL: Immunofluorescence
MannR: Mannose receptor
VOI: Volume of interest
%ID/g: Injected dose per gram
L_OlfB, R_OlfB: Left and right olfactory bulb
L_PFC, R_PFC: Left and right prefrontal cortex
L_Front, R_Front: Left and right frontal cortex
L_OFC, R_OFC: Left and right orbitofrontal cortex
L_Cg, R_Cg: Left and right cingulate cortex
L_MC, R_MC: Left and right motor cortex
L_SSC, R_SSC: Left and right somatosensory cortex
L_S, R_S: Left and right striatum
L_AntHip, R_AntHip: Left and right anterior hippocampus
L_PostHip, R_PostHip: Left and right posterior hippocampus
L_Tha, R_Tha: Left and right thalamus
L_HTha, R_HTha: Left and right hypothalamus
L_MB, R_MB: Left and right midbrain
L_CRB, R_CRB: Left and right cerebellum
FDR: False discovery rate
SEM: Standard error of the mean

## References

[1] Walker FO (2007). Huntington’s disease. Lancet.

[2] Cowan CM, Raymond LA (2006). Selective neuronal degeneration in Huntington’s disease. Curr Top Dev Biol.

[3] Khoshnan A, Ko J, Watkin EE, Paige LA, Reinhart PH, Patterson PH (2004). Activation of the IκB kinase complex and nuclear factor-κB contributes to mutant Huntingtin neurotoxicity. J Neurosci.

[4] Palpagama TH, Waldvogel HJ, Faull RLM, Kwakowsky A (2019). The role of microglia and astrocytes in Huntington’s Disease. Front Mol Neurosci.

[5] Sapp E, Kegel KB, Aronin N (2001). Early and progressive accumulation of reactive microglia in the Huntington disease brain. J Neuropathol Exp Neurol.

[6] Boussicault L, Hérard AS, Calingasan N (2014). Impaired brain energy metabolism in the BACHD mouse model of Huntington’s disease: critical role of astrocyte-neuron interactions. J Cereb Blood Flow Metab.

[7] Hong Y, Zhao T, Li XJ, Li S (2016). Mutant huntingtin impairs BDNF release from astrocytes by disrupting conversion of Rab3a-GTP into Rab3a-GDP. J Neurosci.

[8] Rebec G (2013). Dysregulation of corticostriatal ascorbate release and glutamate uptake in transgenic models of huntington’s disease. Antioxid Redox Signal.

[9] Simmons DA, Casale M, Alcon B, Pham NHA, Narayan N, Lynch G (2007). Ferritin accumulation in dystrophic microglia is an early event in the development of Huntington’s disease. Glia.

[10] Tai YF, Pavese N, Gerhard A (2007). Microglial activation in presymptomatic Huntington’s disease gene carriers. Brain.

[11] Pavese N, Gerhard A, Tai YF (2006). Microglial activation correlates with severity in Huntington disease: a clinical and PET study. Neurology.

[12] Amlani S, Nadarajah T, McIvor RA (2011). Montelukast for the treatment of asthma in the adult population. Expert Opin Pharmacother.

[13] Reiss TF, Altman LC, Chervinsky P (1996). Effects of montelukast (MK-0476), a new potent cysteinyl leukotriene (LTD4) receptor antagonist, in patients with chronic asthma. J Allergy Clin Immunol.

[14] Trinh HKT, Lee SH, Cao TBT, Park HS (2019). Asthma pharmacotherapy: an update on leukotriene treatments. Expert Rev Respir Med.

[15] Michael J, Marschallinger J, Aigner L (2019). The leukotriene signaling pathway: a druggable target in Alzheimer’s disease. Drug Discov Today.

[16] Tang SS, Wang XY, Hong H (2013). Leukotriene D4 induces cognitive impairment through enhancement of CysLT1R-mediated amyloid-β generation in mice. Neuropharmacology.

[17] Yu XB, Dong RR, Wang H (2016). Knockdown of hippocampal cysteinyl leukotriene receptor 1 prevents depressive behavior and neuroinflammation induced by chronic mild stress in mice. Psychopharmacology.

[18] Zhang WP, Hu H, Zhang L (2004). Expression of cysteinyl leukotriene receptor 1 in human traumatic brain injury and brain tumors. Neurosci Lett.

[19] Fang SH, Wei EQ, Zhou Y (2006). Increased expression of cysteinyl leukotriene receptor-1 in the brain mediates neuronal damage and astrogliosis after focal cerebral ischemia in rats. Neuroscience.

[20] Farias S, Frey LC, Murphy RC, Heidenreich KA (2009). Injury-related production of cysteinyl leukotrienes contributes to brain damage following experimental traumatic brain injury. J Neurotrauma.

[21] Chinnici CM, Yao Y, Praticò D (2007). The 5-lipoxygenase enzymatic pathway in the mouse brain: young versus old. Neurobiol Aging.

[22] Ghosh A, Chen F, Thakur A, Hong H (2016). Cysteinyl leukotrienes and their receptors: emerging therapeutic targets in central nervous system disorders. CNS Neurosci Ther.

[23] Lenz QF, Arroyo DS, Temp FR (2014). Cysteinyl leukotriene receptor (CysLT) antagonists decrease pentylenetetrazol-induced seizures and blood-brain barrier dysfunction. Neuroscience.

[24] Huber C, Marschallinger J, Tempfer H (2011). Inhibition of leukotriene receptors boosts neural progenitor proliferation. Cell Physiol Biochem.

[25] Gelosa P, Bonfanti E, Castiglioni L (2019). Improvement of fiber connectivity and functional recovery after stroke by montelukast, an available and safe anti-asthmatic drug. Pharmacol Res.

[26] Kumar A, Prakash A, Pahwa D, Mishra J (2012). Montelukast potentiates the protective effect of rofecoxib against kainic acid-induced cognitive dysfunction in rats. Pharmacol Biochem Behav.

[27] Marschallinger J, Schäffner I, Klein B (2015). Structural and functional rejuvenation of the aged brain by an approved anti-asthmatic drug. Nat Commun.

[28] Zhang CT, Lin JR, Wu F (2016). Montelukast ameliorates streptozotocin-induced cognitive impairment and neurotoxicity in mice. Neurotoxicology.

[29] Lai J, Hu M, Wang H (2014). Montelukast targeting the cysteinyl leukotriene receptor 1 ameliorates Aβ1-42-induced memory impairment and neuroinflammatory and apoptotic responses in mice. Neuropharmacology.

[30] Michael J, Zirknitzer J, Unger MS (2021). The leukotriene receptor antagonist montelukast attenuates neuroinflammation and affects cognition in transgenic 5xFAD mice. Int J Mol Sci.

[31] Saad MA, Abdelsalam RM, Kenawy SA, Attia AS (2015). Montelukast, a cysteinyl leukotriene receptor-1 antagonist protects against hippocampal injury induced by transient global cerebral ischemia and reperfusion in rats. Neurochem Res.

[32] Jang H, Kim S, Lee JM, Oh YS, Park SM, Kim SR (2017). Montelukast treatment protects nigral dopaminergic neurons against microglial activation in the 6-hydroxydopamine mouse model of Parkinson’s disease. NeuroReport.

[33] Han B, Zhang YY, Ye ZQ (2021). Montelukast alleviates inflammation in experimental autoimmune encephalomyelitis by altering Th17 differentiation in a mouse model. Immunology.

[34] Beal MF, Ferrante RJ, Swartz KJ, Kowall NW (1991). Chronic quinolinic acid lesions in rats closely resemble Huntington’s disease. J Neurosci.

[35] Moresco RM, Lavazza T, Belloli S (2008). Quinolinic acid induced neurodegeneration in the striatum: a combined in vivo and in vitro analysis of receptor changes and microglia activation. Eur J Nucl Med Mol Imaging.

[36] Beal MF, Kowall NW, Ellison DW, Mazurek MF, Swartz KJ, Martin JB (1986). Replication of the neurochemical characteristics of Huntington's disease by quinolinic acid. Nature.

[37] Guillemin GJ (2012). Quinolinic acid, the inescapable neurotoxin. FEBS J.

[38] Schwarcz R, Guidetti P, Sathyasaikumar K, Muchowski PJ (2010). Of mice, rats and men: revisiting the quinolinic acid hypothesis of Huntington’s disease. Prog Neurobiol.

[39] Guidetti P, Luthi-Carter RE, Augood SJ, Schwarcz R (2004). Neostriatal and cortical quinolinate levels are increased in early grade Huntington’s disease. Neurobiol Dis.

[40] Kalonia H, Kumar P, Kumar A, Nehru B (2010). Protective effect of Montelukast against quinolinic acid/malonic acid induced neurotoxicity: possible behavioral, biochemical, mitochondrial and tumor necrosis factor-α level alterations in rats. Neuroscience.

[41] Wheeler CJ, Seksenyan A, Koronyo Y (2014). T-lymphocyte deficiency exacerbates behavioral deficits in the 6-OHDA unilateral lesion rat model for Parkinson’s disease. J Neurol Neurophysiol..

[42] Paxinos G, Watson C (2005). The rat brain in stereotaxic coordinates.

[43] di Grigoli G, Monterisi C, Belloli S (2015). Radiosynthesis and preliminary biological evaluation of [^18^F]VC701, a radioligand for translocator protein. Mol Imaging.

[44] Mu S, Wang J, Zhou G (2014). Transplantation of induced pluripotent stem cells improves functional recovery in Huntington’s disease rat model. PLoS ONE.

[45] Belloli S, Zanotti L, Murtaj V (2018). ^18^F-VC701-PET and MRI in the in vivo neuroinflammation assessment of a mouse model of multiple sclerosis. J Neuroinflamm.

[46] Schiffer WK, Mirrione MM, Biegon A, Alexoff DL, Patel V, Dewey SL (2006). Serial microPET measures of the metabolic reaction to a microdialysis probe implant. J Neurosci Methods.

[47] Blumenstock S, Dudanova I (2020). Cortical and striatal circuits in Huntington’s Disease. Front Neurosci.

[48] Horwitz B, Duara R, Rapoport SI (1984). Intercorrelations of glucose metabolic rates between brain regions: application to healthy males in a state of reduced sensory input. J Cereb Blood Flow Metab.

[49] Veronese M, Moro L, Arcolin M (2019). Covariance statistics and network analysis of brain PET imaging studies. Sci Rep.

[50] Rubinov M, Sporns O (2010). Complex network measures of brain connectivity: uses and interpretations. Neuroimage.

[51] Besusso D, Schellino R, Boido M (2020). Stem cell-derived human striatal progenitors innervate striatal targets and alleviate sensorimotor deficit in a rat model of Huntington Disease. Stem Cell Reports.

[52] Hobbs NZ, Barnes J, Frost C (2010). Onset and progression of pathologic atrophy in Huntington disease: a longitudinal MR imaging study. Am J Neuroradiol.

[53] Xia M, Wang J, He Y (2013). BrainNet Viewer: a network visualization tool for human brain connectomics. PLoS ONE.

[54] Cosenza-Nashat M, Zhao ML, Suh HS (2009). Expression of the translocator protein of 18 kDa by microglia, macrophages and astrocytes based on immunohistochemical localization in abnormal human brain. Neuropathol Appl Neurobiol.

[55] Lavisse S, Guillermier M, Hérard AS (2012). Reactive astrocytes overexpress TSPO and are detected by TSPO positron emission tomography imaging. J Neurosci.

[56] Franco R, Fernández-Suárez D (2015). Alternatively activated microglia and macrophages in the central nervous system. Prog Neurobiol.

[57] Saijo K, Glass CK (2011). Microglial cell origin and phenotypes in health and disease. Nat Rev Immunol.

[58] Orecchioni M, Ghosheh Y, Pramod AB, Ley K (2019). Macrophage polarization: different gene signatures in M1(LPS+) vs. Classically and M2(LPS-) vs. Alternatively activated macrophages. Front Immunol.

[59] Burudi EM, Riese S, Stahl PD, Régnier-Vigouroux A (1999). Identification and functional characterization of the mannose receptor in astrocytes. Glia.

[60] Amrouni D, Gautier-Sauvigné S, Meiller A (2010). Cerebral and peripheral changes occurring in nitric oxide (NO) synthesis in a rat model of sleeping sickness: Identification of brain iNOS expressing cells. PLoS ONE.

[61] Giraldi-Guimarães A, de Freitas HT, Coelho BDP (2012). Bone marrow mononuclear cells and mannose receptor expression in focal cortical ischemia. Brain Res.

[62] Pannell M, Economopoulos V, Wilson TC (2020). Imaging of translocator protein upregulation is selective for pro-inflammatory polarized astrocytes and microglia. Glia.

[63] Régnier-Vigouroux A (2003). The mannose receptor in the brain. Int Rev Cytol.

[64] Varinthra P, Ganesan K, Huang SP (2021). The 4-(Phenylsulfanyl) butan-2-one improves impaired fear memory retrieval and reduces excessive inflammatory response in triple transgenic Alzheimer’s Disease Mice. Front Aging Neurosci..

[65] Huang XJ, Zhang WP, Li CT (2008). Activation of CysLT receptors induces astrocyte proliferation and death after oxygen-glucose deprivation. Glia.

[66] Ding Q, Wei EQ, Zhang YJ, Zhang WP, Chen Z (2006). Cysteinyl leukotriene receptor 1 is involved in *N*-methyl-d-aspartate- mediated neuronal injury in mice. Acta Pharmacol Sin.

[67] Braune M, Scherf N, Heine C (2021). Involvement of GPR17 in neuronal fibre outgrowth. Int J Mol Sci.

[68] Rees EM, Scahill RI, Hobbs NZ (2013). Longitudinal neuroimaging biomarkers in Huntington’s disease. J Huntingtons Dis.

[69] Magistretti PJ, Pellerin L (1996). The contribution of astrocytes to the ^18^F-2-deoxyglucose signal in PET activation studies. Mol Psychiatry.

[70] Braidy N, Grant R, Adams S, Brew BJ, Guillemin GJ (2009). Mechanism for quinolinic acid cytotoxicity in human astrocytes and neurons. Neurotox Res.

[71] Polyzos AA, Lee DY, Datta R (2019). Metabolic reprogramming in astrocytes distinguishes region-specific neuronal susceptibility in Huntington Mice. Cell Metab.

[72] Im HJ, Hahm J, Kang H (2016). Disrupted brain metabolic connectivity in a 6-OHDA-induced mouse model of Parkinson’s disease examined using persistent homology-based analysis. Sci Rep.

[73] Liang S, Jiang X, Zhang Q (2018). Abnormal metabolic connectivity in rats at the acute stage of ischemic stroke. Neurosci Bull.

[74] Tartaglione AM, Armida M, Potenza RL, Pezzola A, Popoli P, Calamandrei G (2016). Aberrant self-grooming as early marker of motor dysfunction in a rat model of Huntington’s disease. Behav Brain Res.

[75] Scattoni ML, Valanzano A, Popoli P, Pezzola A, Reggio R, Calamandrei G (2004). Progressive behavioural changes in the spatial open-field in the quinolinic acid rat model of Huntington’s disease. Behav Brain Res.

[76] Shenoy SA, Zheng S, Liu W (2022). A novel and accurate full-length HTT mouse model for Huntington’s disease. Elife.

